# Partial anomalous pulmonary venous return after orthotopic heart transplantation case report

**DOI:** 10.1186/s12872-020-01818-1

**Published:** 2021-01-06

**Authors:** Ismael A. Salas de Armas, Manish K. Patel, Bindu Akkanti, Jorge Salazar, Biswajit Kar, Igor D. Gregoric

**Affiliations:** 1 Department of Advanced Cardiopulmonary Therapies and Transplantation, Center for Advanced Heart Failure, The University of Texas Health Science Center at Houston/Memorial Hermann Hospital – Texas Medical Center, 6400 Fannin St., Suite 2350, Houston, TX 77030 USA; 2 Department of Internal Medicine, Divisions of Critical Care, Pulmonary and Sleep, The University of Texas Health Science Center at Houston/Memorial Hermann Hospital – Texas Medical Center, Houston, TX USA; 3 Pediatric Cardiothoracic Surgery, The University of Texas Health Science Center at Houston/Memorial Hermann Hospital – Texas Medical Center, Houston, TX USA

**Keywords:** Partial anomalous pulmonary venous return, Heart transplant, Case report

## Abstract

**Background:**

Partial anomalous pulmonary venous return (PAPVR) is a congenital heart defect. Reports of repair and treatment in pediatric cases have been published, but incidence of PAPVR in adults is not common. To our knowledge, there has not been a diagnosis of left-sided PAPVR after a heart transplant an in adult patient.

**Case presentation:**

A 62-year-old patient with ischemic cardiomyopathy and systolic heart failure underwent orthotopic heart transplantation. The immediate post-operative course was remarkable for an elevated cardiac index and pulmonary artery pressures as well as decreased systemic vascular resistance. The post-operative echocardiogram did not reveal an intra-cardiac shunt. However, computed tomographic angiography (CTA) showed a left superior pulmonary vein draining into the innominate vein. Operative repair of the left superior pulmonary venous connection to the left atrial appendage was completed under cardiopulmonary bypass with beating heart. Her hemodynamics improved immediately, and she had an unremarkable postoperative course.

**Conclusions:**

While uncommon, any patient with a high cardiac output and abnormal hemodynamics after heart transplant should be evaluated for the existence of a shunt. While not a part of all traditional preoperative imaging protocols, a chest CTA should be considered if PAPVR is suspected as it can both diagnose the condition and enable a plot of the corrective course of surgical action.

## Background

Partial anomalous pulmonary venous return (PAPVR) is a congenital abnormality where a single, or some, pulmonary veins fail to establish connection to the left atrium. As a result, one or more pulmonary veins drain anomalously to the right heart through the right atrium (intracardiac) or a systemic vein (extracardiac) [1]. Most commonly, the right-side pulmonary veins are involved in this abnormal connection in children, with the coexistence of an atrial septal defect [2]. Left-sided PAPVR is rarer and accounts for 10% of all reported PAPVR cases [3]. Adults with undiagnosed PAPVR may have an intact atrial septum and no pulmonary vein obstruction, resulting in minimal symptoms until an additional insult occurs to the heart. Because it is uncommon, the diagnosis of PAPVR may be overlooked. Herein, we report a unique case of adult, left-sided PAPVR that was identified after an orthotopic heart transplant (OHT).

### Case presentation

A 62-year-old female underwent a diagnostic left heart catheterization in 2012. The procedure was complicated by a left main coronary artery dissection requiring an emergent coronary artery bypass graft (CABG) of the left anterior descending coronary artery and the obtuse marginal artery. Unfortunately, the dissection of her left main coronary artery resulted in the worsening of the patient’s heart failure symptoms despite the emergent CABG. Since the patients’ ejection fraction after CABG never improved, she transferred to our advanced heart failure clinic. Over two years, multiple right heart catheterizations (RHCs) were completed; Fick method calculations consistently demonstrated a normal cardiac index. However, the patient experienced worsening symptoms. Once she was profoundly symptomatic, her case was presented at the medical review board, and she was approved for an OHT. Her transplant was uneventful; however, during surgery an abnormal amount of blood was noted draining from the pulmonary veins. Her initial 24-h post-operative course was remarkable for a high cardiac index and right ventricular (RV) cardiac output, a low systemic vascular resistance, and an elevated pulmonary pressure (Table 1).

**Table 1:** Cardiac measures of case over time

	RA pressure (mm Hg)	RV pressure (mm Hg)	PA pressure (mm Hg)	PA saturation (%)	PCWP (mm Hg)	SVR in (dynes/seconds/cm^−5^/WU	Fick cardiac output/cardiac index	Arterial saturation (%)	Hemoglobin
20 months prior to transplant	2	34/1	36/11	71.5	10	1092/13.6	5.93/3.48	93	12.3
16 months prior to transplant	12	63/8	64/27	68.3	38	1490/18.6	4.08/2.43	94	14.7
POD-30	11	72/10	68/33	73%	33	1576/19.7	4.11/2.54 TD: 3.87/2.39	99	14.2
POD-12	4	33/5	33/10	65%	15	1147/14.3	5.02/2.96 TD: 5.07/2.99	92	14.2
POD-3	14	65/11	65/35	65%	37	1222/15.2	5.17/2.99 TD: 2.78/1.60	98	9.0
Transplant surgery (POD 0)	11		32/14	75%	12	613/7.6	9.0/5.1	98	10.0
Repair surgery POD 2	9		30/14	65%	10	1445/18	5.7/3.2	98	10.2
POD 17	10	36/10	34/21	60.2	19	1301/16	5.1/2.88	96	8.9
POD 60	4	21/2	24/12	71%	8	1136/14	5.7/3.5	93	11.9

*RA* right atrial, *RV* right ventricle, *PA* pulmonary artery, *PCWP* pulmonary capillary wedge pressure, *POD* post-operative day

**Fig. 1:**
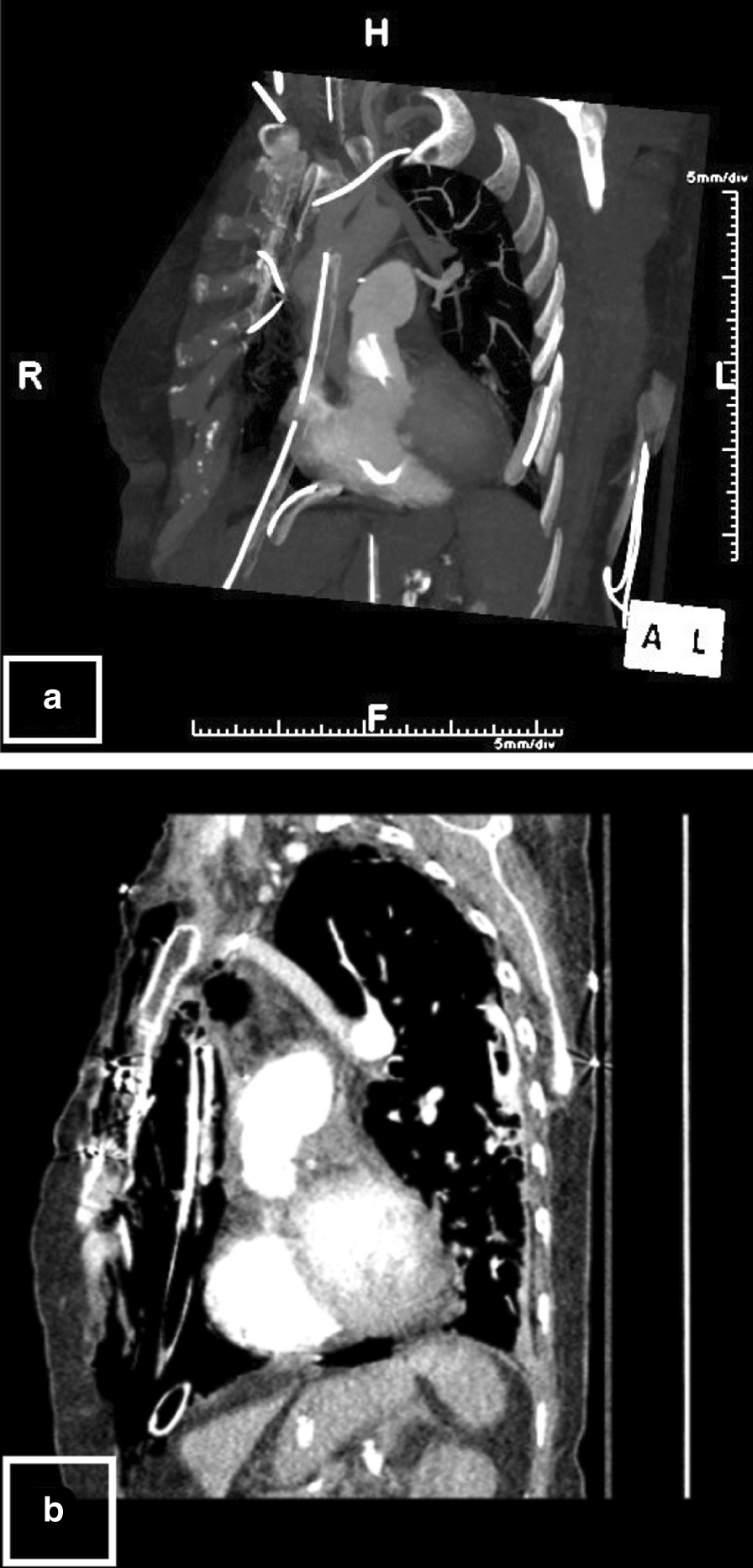
**a** The computed tomography (CT) image (sagittal view) shows the left superior pulmonary vein draining into the innominate vein. **b** CT Image with an alternate contrasting

While a shunt was suspected, an iatrogenic shunt was ruled out via bubble echocardiography. However, computed tomography (CT) scans revealed an abnormal left superior pulmonary vein draining into the innominate vein (Fig. 1). The CTA demonstrated the presence of an extracardiac shunt in the form of PAPVR.

**Fig. 2:**
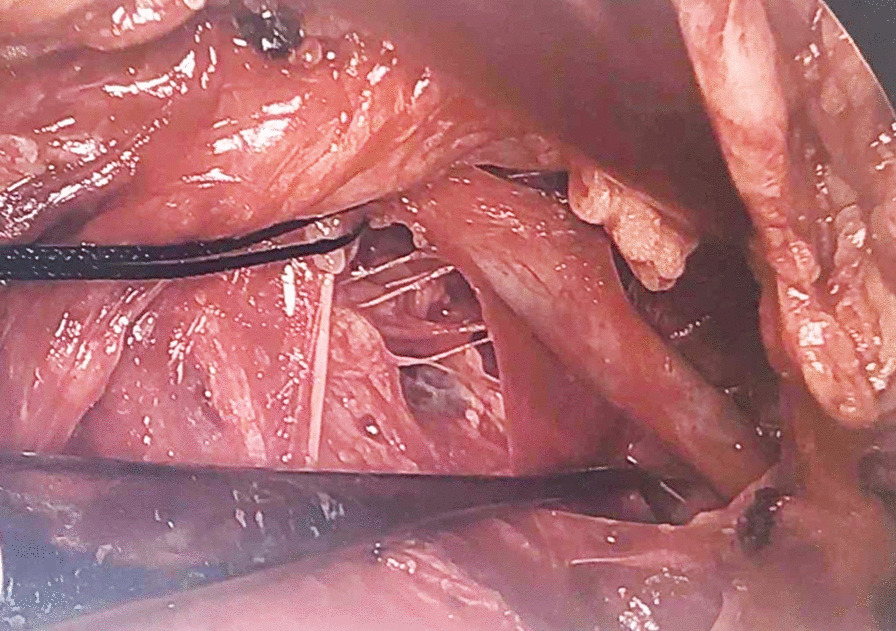
The venous connection between the left superior pulmonary vein and the innominate vein is shown. A branch was ligated with silk suture

**Fig. 3:**
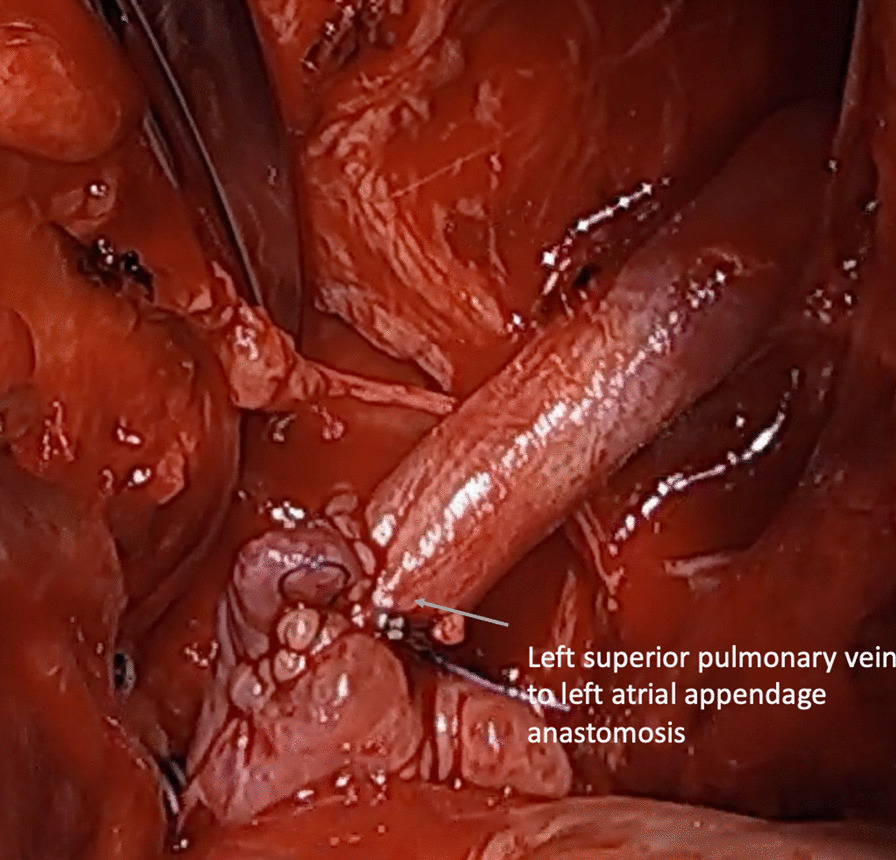
The anastomosis between the left superior pulmonary vein and the left atrial appendage is visualized

After a multidisciplinary decision to pursue operative repair, the chest was re-opened. The left superior pulmonary vein (LSPV) was identified and visualized. A careful dissection was initiated from the pulmonary hilum toward the innominate vein outside the pericardium (Fig. 2). The venous connection was completely mobilized, a tributary branch was ligated, and the vein was transected at the innominate level. After mobilizing the vein, an end-to-side anastomosis was made between the LSPV and the donor heart left atrial appendage while under cardiopulmonary bypass with beating heart (Fig. 3). The heart and lung machine was used to avoid hemodynamic instability during the exposure of the left atrial appendage for the anastomosis. After the anastomosis and hemostasis, the patient was weaned from cardiopulmonary bypass and no inotropic support was required. The cannulas were removed after protamine was administered. The chest was closed in standard fashion, and the patient recovered in the intensive care unit. The patient was discharged home on Day 15.

### Discussion and conclusions

Imaging studies contend that PAPVR exists in about 0.1–0.2% in the general adult population [4]. However, an autopsy series found that PAPVR is found in 0.4% of cases [5]. In contrast to symptomatic pediatric cases, adults with undiagnosed PAPVR have a silent clinical course or mild symptoms. Typically, these patients have intact atrial septum and no pulmonary vein obstruction. If found, surgical repair is possible. Majdalany and colleagues reported the identification of PAPVR in 43 adult patients; of those, 28 elected for operative repair in the setting of isolated pulmonary venous connection [2]. Surgical morbidity and mortality were low in their series and most improved the right ventricle size and pulmonary pressures [2].

The systemic venous connection found in adult PAPVR patients is a left-to-right shunt that can lead to high RV cardiac output and heart failure. These patients are at risk for developing pulmonary arterial hypertension due to chronic volume overload. Further, if an additional insult occurs to the heart, symptoms and complications from PAPVR result.

In the presented case, the patient initially tolerated the amount of left-to-right shunt throughout her adulthood. Symptoms of PAPVR began after the coronary dissection and continued to worsen for which she underwent OHT. Based upon the hemodynamics, a shunt was expected; however, echocardiography imaging was negative for intracardial shunt. Importantly, the use of CTA confirmed the PAPVR diagnosis. The documentation of a LSPV draining into the innominate vein was crucial; thus, CTA is the gold-standard imaging tool to diagnose PAPVR.

This clinical entity is not common, and few guidelines for newly diagnosed congenital heart disease in adults older than 40 years old have been published [6]. Surgical repair was relatively straightforward and re-established the natural connection between the anomalous vein and the left atrial appendage.

The decision of immediate surgical repair is paramount in this case. The transplanted right ventricle is not conditioned for pulmonary artery over-circulation and right ventricular load, as this can lead to early heart failure and, potentially, decreased life expectancy.

In 2015, Ksela and colleagues reported the successful completion of an OHT with concomitant repair of PAPVR [7]; however, no other published literature on the repair of PAPVR in adult OHT patients is available. Thus, while infrequent, the transplant community should consider PAPVR if a patient demonstrates high RV cardiac output and irregular hemodynamics with no intracardiac shunt.

## Data Availability

The dataset used for this case report is available from the corresponding author on reasonable request. All data was obtained from the patient’s electronic medical record.

## Abbreviations

CABG: Coronary artery bypass graft
CT: Computed tomography
HFrEF: Heart failure with a reduced ejection fraction
LSPV: Left superior pulmonary vein
OHT: Orthotopic heart transplantation
PAPVR: Partial anomalous pulmonary venous return
